# Lessons and long-term implications of the covid-19 response in Denmark from a resilience perspective: Karsten Vrangbæk

**DOI:** 10.1093/eurpub/ckac129.349

**Published:** 2022-10-25

**Authors:** K Vrangbæk

**Affiliations:** Department of Public Health, University of Copenhagen, Copenhagen, Denmark

## Abstract

The paper focuses on crisis responses and resilience within hospitals. The study is based on structured expert interviews with medical directors in selected hospital departments in two of the five regions of Denmark and primary care physicians in the same regions. We investigate stage 3 (Shock impact and management) and stage 4 (Recovery and learning) within hospital organizations using Denmark as case country, and we pay particular attention to issues of “organisational learning”, “purchasing flexibility and reallocation of funding”, “distribution of human and physical resources” and “motivated and well-supported workforce”. Particular attention is paid to care for patients with chronic care needs and lessons for the long-term resilience building in the health system. The study highlights strategic choices and lessons for the long-term resilience within hospitals. It demonstrates, how the initial strategy of organizing specific COVID-19 response units was abandoned relatively early, as it appeared more efficient to integrate COVID-19 patients in the regular specialized department structure. Emergency wards experienced increasing pressure during the pandemic as primary care clinics were referring (too) many patients suspected of COVID-19. This raises questions about capacity and relations between primary and specialized care in crisis situations. Management of human resources is crucial. While the initial phases of the pandemic response were characterized by flexibility and “team-spirit”, there has been a negative long-term impact particularly among the nursing staff, where burnouts and attrition are major issues. Pandemic crises place significant strain on health systems and personnel. This raises issues about communication of strategies and principles for organizing efforts. The Danish health system managed the crisis adequately, but there are also lessons that should be learned regarding long-term implications and preparedness for future crises.

